# Bringing the group back in: Social class and resistance in adolescent smoking

**DOI:** 10.1111/1467-9566.13858

**Published:** 2024-10-22

**Authors:** Olivia McEvoy, Richard Layte

**Affiliations:** ^1^ Department of Sociology Trinity College Dublin Dublin 2 Ireland

**Keywords:** adolescence, group behaviour, health inequalities, smoking, social determinants, social resistance, social theory

## Abstract

Absolute prevalence of tobacco smoking has fallen in recent decades but inequalities by socioeconomic position (SEP) persist. Adolescence is a critical period for smoking initiation and habits formed during this period likely continue into adulthood. Explanations for inequalities in adolescent smoking have tended to focus on individualistic theories based on differentials in knowledge and psychology. These have been criticised for their blindness to processes of social stratification and social context that influence smoking behaviours. Based on previous social theories, we put forward, and test empirically, two potential structural explanations for inequalities in smoking, using nationally representative longitudinal cohort data on 6039 Irish young people aged 9–18 years. Descriptive analyses confirmed the adverse SEP gradient in smoking prevalence as well as SEP gradients in variables representing individual‐level characteristics and structural‐level explanations. Despite lower self‐esteem being associated with a higher likelihood of smoking, there was no significant indirect pathway between SEP and smoking via self‐esteem. Path analyses found that differentials in exposure to parental smoking and levels of oppositional values mediate the relationship between SEP and smoking. Our results favour structural and group‐based explanations for inequalities, that is, the ‘smoking exposure’ and ‘social resistance’ models, over explanations based on individual psychology.

## INTRODUCTION AND BACKGROUND

The finding that morbidity and mortality risk are inversely related to the socioeconomic position of individuals, within countries, is one of the most consistent findings in sociological and epidemiological research (Stringhini et al., 2017). Although the nature of the relationship varies depending on the measure of socioeconomic position used (i.e. income, education and occupation; henceforth socioeconomic position (SEP)), the risk of poor health and mortality increases with disadvantage and, for this reason, the relationship has come to be known as the ‘social gradient in health’ (Tanaka et al., 2019). SEP does not influence health directly and researchers continue to test potential mechanisms through which it ‘gets under the skin’ (Blane et al., 2013). The patterning of health by social determinants (i.e. SEP) can be explained, in part, by differentials in harmful health behaviours by SEP. Health behaviours, such as smoking, have been shown to cluster according to SEP and increase the risk of mortality from non‐communicable diseases (Petrovic et al., 2018).

Smoking is the leading cause of preventable death globally and the single largest avoidable health risk (WHO, 2022). It also contributes to substantial health‐related costs to society through smoking‐attributable medical expenditures and lost productivity (Goodchild et al., 2018). Particular attention, in terms of policy intervention, is paid to youth smoking given that 93% of smokers take up smoking before they turn 24 (European Commission, 2024). Adolescence is a ‘critical period’ for the formation of risky health behaviours (Inchley et al., 2020; Viner et al., 2015). For this reason, research exploring potential causal factors for persistent smoking among adolescents is key if we are to eliminate the health disparities related to smoking that are evident in adult populations. Whilst absolute rates of smoking have been falling in recent decades (OECD, 2021; Staff et al., 2018), reductions have been larger among more socioeconomically advantaged groups. The resulting widening socioeconomic gradient in smoking, as overall population rates decline, suggests that public health initiatives designed to reduce smoking rates have had little effect on the most disadvantaged members of society (Hiscock et al., 2012; Lawlor et al., 2003).

Less is known about *why* smoking varies across social groups in this structured manner, which is the focus of this paper. The dominant focus of social epidemiology on the immediate causes of disease has neglected to consider the larger social forces that expose individuals to these health behaviours in the first place, an observation initially highlighted in the Black report (1982).

### Individual‐level explanations for inequalities in smoking

For decades, literature on health promotion largely focused on the role of individual factors in shaping the probability of adopting risky health behaviours and by extension for explaining differentials in behaviours across SEP groups (Cockerham, 2005; Marmot, 2018). These *individual‐level explanations* come in three forms. First, the ‘health knowledge’ model views risky health behaviours as the outcome of poor information and argues that smoking is adopted or maintained because individuals do not know about the negative effects (Layte & Whelan, 2009). The ‘rational choice’ model, on the other hand, asserts that people can know about the risks involved but still choose to smoke because they decide to trade off the current pleasure that can be derived from smoking against the more uncertain future health risks that may emerge, similar to ‘hyperbolic discounting’ (Ainslie & Haslam, 1992) or ‘countervailing mechanisms’ (Phelan et al., 2010). This model prioritises individual agency and the individual’s evaluation of short‐ and long‐term incentives and acknowledges the long‐standing tradition within sociology and anthropology that behaviours that may be difficult to understand are rational within the particular contexts in which they occur. The third model, the ‘psychological’ model (Delaney & Doyle, 2012; Layte & Whelan, 2009) holds that individuals can know about the risks that a behaviour poses, and may even want to stop, but smoke nonetheless because of certain psychological factors. For example, low or decreasing adolescent self‐efficacy has been shown to predict smoking initiation (Lawrance & Rubinson, 1986; Van De Ven et al., 2007) largely through its negative influence on the young person’s capacity not to smoke among smoking friends (Hiemstra et al., 2011), and there is a well‐established literature on the role of low self‐esteem in adolescent smoking initiation (Khosravi et al., 2016).

### Structural‐level explanations for inequalities in smoking

Whilst there is certainly evidence supporting these three models of adolescent smoking, it could be argued that in focusing on the role of individual deficits, biases and psychological traits, each misses the important role that structural factors play in the initiation and maintenance of smoking. In the early stages of cigarette diffusion, there was a strong positive relationship between social advantage and smoking, but in the later stages of cigarette diffusion the relationship reversed (Pampel, 2005). This changing relationship between SEP and smoking, overtime, suggests that social forces play a role in explaining the variation in smoking across social groups. More structurally orientated researchers have sought to unpack the processes through which social stratification and social context influence health behaviours at the individual‐level (McCartney et al., 2013).

For example, Cockerham (2005) argued that the clustering of smoking according to SEP is not the uncoordinated response of disconnected individuals but the patterned response of social groups to the constraints of their external environments, drawing on Weber’s (1946) concept of ‘Lebenschancen’ (life chances). Moreover, Bartley (2016) employs the work of Bourdieu (1989) to unpack the symbolic dimensions of smoking by arguing that smoking is a strategy adopted by individuals to display group membership and distance themselves from other non‐smoking groups. In this way, smoking acts as a consumption practice for identity and status. This work provides us with the broad sociological framework for the structural explanations put forward in this paper.

The first *structural‐level explanation* put forward by this paper is based on social learning theory and social network theory (Bandura & Walters, 1977; Granovetter, 1973). Variation in smoking according to SEP suggests subcultural differences in the content of what is learnt or reinforced within the young person’s ‘micro‐system’, that is, parents, other household members and friends (Bronfenbrenner, 1977). Since smoking has been shown to be more prevalent among those with disadvantaged SEP, this would suggest that young people from disadvantaged backgrounds are exposed to more smoking in their social environment, compared to their peers from more advantaged backgrounds. Smoking has been shown to be more likely among parents who are younger, less educated and have more financial difficulties (Hitchman et al., 2014; Leonardi‐Bee et al., 2011). The process through which this proximity may lead to smoking is, however, disputed. Social learning theory postulates that smokers learn to smoke by the same processes that non‐smokers learn not to smoke by the example of parents. Alternatively, according to Harris (2000, 1998, 1995), ‘socialisation’ processes occur when the young person inherits a peer network and ‘shared culture’ from their parent’s network, which demands the same behaviours from the young person as it did from their parents, that is, smoking. Young people who have a high likelihood of smoking may seek out peers who already smoke, but it is also possible that exposure to smoking from peers leads to smoking through socialisation effects. A recent systematic review of 40 studies of adolescent substance use found support for peer selection, with relatively few studies reporting socialisation effects (Henneberger et al., 2021).

The second structural‐level explanation put forward by this paper is based on the work of Factor and colleagues and is a group‐based explanation for social inequalities in adolescent smoking (2011, 2013). The authors observed that, within societies, non‐dominant minority groups exhibit higher rates of risky health behaviours, compared to the dominant majority group. They argue that this pattern emerges as a result of experiences of discrimination, which makes non‐dominant groups feel alienated or rejected from the larger society. By engaging in risky behaviours, such as smoking, the non‐dominant groups ‘express their willingness and ability to defy… the dominant group’ (p. 1293). Their theory builds on Scott’s (1990) theory of ‘everyday resistance,’ which seeks to understand the ways in which marginalised groups defy dominant groups and ‘the system,’ with small acts of resistance, in the context of peasant societies, some of which may improve their standard of living, but many of which provide symbolic empowerment and take back agency. Johnson and Hoffmann (2000) compared smoking initiation between members of minority groups attending ‘minority’ schools (where there is no opposing dominant majority) and members attending majority schools (where there is an opposing dominant majority). Although minority schools have been linked with low educational achievement and high risk of dropout, their results indicated that attendance results in a reduced risk of smoking among minority adolescents, supporting our argument for smoking as a ‘resistance’ behaviour. Lister (2015), also using Scott’s theory, has proposed a similar characterisation of actions among non‐dominant groups, particularly people in poverty, as ‘getting back at’ behaviours.

As well as ‘getting back at,’ smoking can be used to differentiate minority groups from the dominant majority group by asserting an oppositional social identity. In the same way, adopting smoking was initially seen as a form of distinction between those who had the resources to adopt it and those who did not (Elias, 1939). Factor et al. (2011), in the context of racial differences in risky behaviours in the US, observed that non‐dominant groups feel a pressure not to embrace the attitudes and behaviours identified with the dominant group, what they term ‘acting white,’ even though they understand that these behaviours may have harmful consequences for their health. Similar processes of group identity have been central to the educational resistance literature, most famously in the work of Willis (1977) on the activities of the ‘lads’ in a British ‘working‐class’ boy’s school. The ‘lads’ created their own oppositional subculture, which exerted maximum distinction from institutional conformity. This included their own take on the school uniform, getting out of doing homework and having ‘a crafty smoke’ during the school day (p. 23). Their ostracism of those who strove for academic success had the effect of reproducing their family’s educational disadvantage. In this way, there is a ‘complexity and even irrationality within resistance’ (Johansson & Vinthagen, 2016). This argument refers to the recognised ‘dark side’ of social capital, where communities can reject certain behaviours that may be deemed as aligned with the dominant majority group (Schreuders et al., 2017).

It could be argued that the central theoretical problem of sociology since Durkheim (1895) has been the identification of the processes through which social structures shape individual behaviour and the role of individual agency in this. This study provides a concrete example of one such process that can be tested empirically. This article first quantifies the social gradient in smoking in a representative sample of young people from Ireland. The paper then uses path analysis to empirically examine the contribution of an individual‐level theory and structural‐level theory for the social patterning of smoking. Crucially, our approach tests whether the same pathways operate differently depending on the socioeconomic subgroup.

## DATA AND METHODS

### Data

This study used data from Growing Up in Ireland (GUI), a nationally representative cohort study of children living in the Republic of Ireland. The GUI commenced when participants were aged nine with 8568 participants. Data collection for wave one took place between September 2007 and June 2008 (henceforth 2007/2008). Data was subsequently collected when children were aged 13 (between August 2011 and February 2012, henceforth 2011/2012) with an 88% retention rate (*n* = 7525), aged 17/18 (between November 2015 and September 2016, henceforth 2015/2016) with a 73% retention rate (*n* = 6216) and aged 20 (between August 2018 and June 2019, henceforth 2018/2019) with a 61% retention rate (*n* = 5190). The sample was selected through a two‐stage clustered sampling method within the Irish primary school system, with the school as the primary sampling unit and the age eligible children attending the school as the secondary sampling units. Probability proportionate to size sampling was used to select a representative sample of 910 primary schools (82% response rate) with 8568 (57% response rate) families from these schools agreeing to participate. Interviews were carried out with children and parents at home and with teachers and principals at school by trained interviewers.

Levels of missing data across variables were low, on average, but reached 3.7% on some variables (see Table S1 in appendices). Survey attrition was, in part, due to the lack of retention of more disadvantaged participants, meaning there were systematic differences between missing and complete data according to socio‐demographic variables observed in our dataset. This had the potential to bias our analysis, and so missing values were imputed for analysis using multiple imputation by chained equations to produce 10 imputed datasets, using the Rubin method (Rubin, 1987) as implemented in STATA 15. All of the variables in table one (see Supporting Information S1: Appendix) were included in the imputation process with the exception of the smoking status (dependent variable) and family SEP (independent variable). The data were also reweighted for analyses to take account of the original sample error and subsequent attrition using a range of factors and a minimum distance algorithm. The final imputed and reweighted sample (*n* = 6039) is representative of young people who were residing in Ireland at age nine in 2006 and who continued to live in Ireland at age 17/18 in 2016.

### Measures

#### Dependent variable—Smoking status at age 17/18

Smoking status was self‐reported by the young person at wave three, when they were aged 17 or 18 years (henceforth 17/18): ‘have you ever smoked a cigarette?’ (yes/no) and subsequently ‘which of the following best describes you?’ (only ever tried smoking once or twice/used to smoke but not now/smoke occasionally/smoke daily/do not smoke). The answer categories were recoded into ‘smokers’ and ‘non‐smokers,’ with respondents who answered ‘no’ to the first question and ‘only ever tried smoking once or twice,’ ‘used to smoke but not now’ and ‘do not smoke’ being classified as non‐smokers. The proportion of occasional smokers was too low to consider separately for the proposed subgroup analysis (discussed further in the limitation section of this paper). Due to the sensitive nature of the questions (smoking is illegal under the age of 16 in Ireland and parents may not be aware of the participant’s smoking), the questions were administered on a supplemental self‐complete questionnaire, and the young person was assured of confidentiality. The young person, at wave three, also reported what age they were when they initiated smoking and the answers ranged from aged 12 to 18 years. This variable was used in a supplemental analysis.

#### Treatment variable—Family SEP

SEP was assigned to the participant and their household based on parental occupation at wave one (age 9). For two‐parent families in which both partners were economically active outside the home, the family’s SEP was assigned using a ‘dominance’ procedure with family SEP equal to that of the higher of the two parents. A fivefold classification of SEP (professionals and higher management/lower management or technical worker/non‐manual worker/skilled manual/semi or unskilled manual) based on the Standard Occupational Classification of the Central Statistics Office (CSO, 2023) was applied.

### Mediating variables

#### Psychological

Self‐efficacy was measured at wave three (age 17/18) by asking the young person about their belief in their ability to succeed in specific situations and to accomplish tasks. The scale contained six items relating to general self‐efficacy (e.g. ‘If I cannot do a job the first time I keep trying until I can’) and social self‐efficacy (e.g. ‘I find it easy to make new friends’) with four answer categories ranging from ‘strongly agree’ to ‘strongly disagree.’ The scores on the items relating to general and social efficacy were reverse coded where necessary and summed to produce an overall self‐efficacy score, which ranged from zero to 24. Higher scores were indicative of greater self‐efficacy. Self‐efficacy has been found to be strongly predictive of health behaviour initiation and cessation (Lawrance & Rubinson, 1986; Van De Ven et al., 2007). The scale used in the present study was adapted from the Sherer et al. (1982) measure by researchers on the ESRC 16–19 initiative research programme. Internal reliability for this scale at age 17/18 of GUI was *α* = 0.72 (Murphy et al., 2019).

Self‐esteem was measured using the Rosenberg Self‐Esteem scale (Rosenberg, 1965). At wave three, the young person was asked to rate six items (e.g. ‘on the whole, I am satisfied with myself’ and ‘I certainly feel useless at times’) on a four‐point scale ranging from ‘strongly disagree’ to ‘strongly agree.’ The scores on six items were combined, and reverse coded where necessary, to get an overall self‐esteem score, which ranged from zero to 18. Higher scores were indicative of higher levels of self‐esteem. Previous research has highlighted the role of low self‐esteem on the initiation of risky health behaviours (Bozzini et al., 2020). The scale is the most widely used and well‐validated measure of self‐esteem (Robins et al., 2001).

### Smoking exposure

At wave two (age 13), the primary caregiver (PCG; the mother in 98% of the sample) reported on their own smoking status: ‘do you currently smoke daily, occasionally or not at all?’ The three categories (daily/occasionally/not at all) were recoded into two categories ‘parental smoking’ and ‘no parental smoking’ where both daily and occasional smokers were considered smoking parents. The PCG also reported on the number of smokers in the young person’s primary residence: ‘including yourself, how many members of the household smoke?’ This question allowed us to distinguish between households where only the PCG smoked and households with additional smokers.

At wave three (age 17/18), the young person was asked: ‘How many of your regular friends do or have ever smoked cigarettes?’ There were four possible response categories (none/a few/some/most/all). The four response categories were recoded into two categories which indicated (1) ‘none/few friends smoke’ or (2) ‘most/all friends smoke.’ This question was asked as part of the supplemental self‐complete questionnaire where administration was designed with sensitive questions in mind.

### Social‐resistance

The Everyday Discrimination Scale (EDS) was administered at wave three in the supplemental self‐complete sensitive questionnaire to the young person directly. The EDS was constructed using five statements about how often participants felt they had experienced various forms of interpersonal mistreatment (e.g. ‘being threatened or harassed,’ ‘receiving less courtesies or respect than others’). The young person indicated the frequency of these acts on a six‐point scale from ‘never’ to ‘almost every day.’ A total discrimination score was generated from the sum of all five items. Higher scores are indicative of more frequent discrimination. The scores range from zero to five. This five‐item scale was adapted from the original nine‐item version of the EDS (Williams et al., 1997). The scale was found to have good internal reliability at age 17/18 of GUI with *α* = 0.74 (Murphy et al., 2019).

The extent to which the young person had developed oppositional values was measured using a scale from the ESRC 16–19 initiative research programme to measure relationships with authority. The scale was administered at wave three to the young person. The scale contained eight items:

1. It can be okay to do something which is against the law if it is to help a friend.
2. People in authority, like teachers, always pick on me.
3. Most of the rules in places like schools are stupid and petty.
4. School has been a waste of time for me.
5. Defying people in authority is alright if you can get away with it.
6. The police are often unnecessarily brutal to people.
7. If I saw someone make a break in, I would tell the police about it.
8. Most police officers are honest.

The young person indicated their level of agreement with the statements on a four‐point scale (strongly agree/agree/disagree/strongly disagree). Scores on the eight items were reverse coded where necessary and summed to produce an overall score ranging from zero to 32. Higher scores are indicative of more opposition to authority. For some descriptive statistics, it was necessary to split the responses into tertiles: ‘low,’ ‘medium’ and ‘high’ levels of oppositional values. The Cronbach alpha value for the scale at age 17/18 of GUI was *α* = 0.72 (Murphy et al., 2019).

### Control variables

At wave one, basic descriptive information about the young person was captured including sex (male/female) and the young person’s personality. It was not possible to split the sample by gender, in order to observe unique pathways for males and females, due to the small number of smokers at wave three. Since previous research suggests that smoking is significantly associated with certain personality traits (Vollrath et al., 1999), the 10 Item Personality Inventory (TIPI) was used to measure aspects of the young person’s personality, at wave three, as reported by the young person. TIPI consists of five subscales: agreeableness, extraversion, conscientiousness, emotional stability and openness to experience (Gosling et al., 2003). Classifying individual differences in personality into these five broad empirically derived domains is one of the most widely used and extensively researched models of personality. Each personality dimension was rated on a seven‐point scale with answer categories ranging from disagree strongly to agree strongly, where strongly agree indicates a stronger presence of the respective personality trait. Gosling et al. (2003) noted the scale has good test–retest reliability (*r* = 0.72) and reported convergent correlations with the personality scale: the ‘Big‐Five Inventory.’ Furthermore, it was not hypothesised that gender or personality mediated the relationship between SEP and smoking, so they were included as a control as opposed to mediating variables in the analysis.

### Analysis strategy

Descriptive statistics were computed to understand the sample characteristics and patterns according to SEP and smoking status. Cross‐tabulation chi‐squared (*χ* ^2^) tests, with 95% CIs, were performed to examine the relationship between SEP and smoking, as well as SEP and gender (control variable), parental, friend’s and household smoking (mediating variables). The association between SEP and self‐esteem, self‐efficacy, everyday discrimination and levels of opposition to authority (mediating variables, continuous) was assessed through one‐way ANOVA analyses. Levene’s test for equality of variance was used to assess that the ANOVA analyses were valid. The associations between smoking status and continuous control (personality) and mediating variables (self‐esteem, self‐efficacy, everyday discrimination and levels of opposition to authority) were assessed by performing independent sample *t*‐tests. The association between smoking and the remaining mediating variables (parental, friend’s and household smoking, binary) were assessed by performing cross‐tabulation chi‐squared (*χ* ^2^) tests with 95% CIs. Results were visually displayed and interpreted using bar chats and line graphs.

This study employed generalised structural equation modelling (GSEM) to test the hypothesised causal pathways by which family SEP (age 9) would contribute to the probability of smoking (age 17/18). GSEM relaxes the constraints of structural equation models (SEM) by allowing for binary modelling of dependent variables. Using GSEM, we fit our models of interest separately allowing us to compare their efficacy before fitting them all together. All variables included in the models were observed variables (none were latent). There are some disadvantages to using GSEM: (1) it does not provide standardised coefficients; therefore, only the significance/non‐significance of coefficients can be used to *compare* the strength of potential pathways, although coefficients can still be interpreted in their own right. (2) The default estimation method in most SEM programs is maximum likelihood; an underlying assumption of this method is a multivariate normality of the observed variables. Our data clearly violates this assumption, as many of our variables are binary and so assume a Bernoulli distribution. Therefore, we examined model fit using summary statistic data. All analyses were carried out on imputed data with appropriate study weights.

Path analysis allows us to examine how the effect of SEP on smoking probability is mediated, by estimating the relationship between SEP and our mediating variables, as well as, the effect of the mediators on smoking probability. If both equations reveal significant effects, we have evidence of a statistically significant mediating pathway. Path analysis cannot demonstrate causality and significant results should be interpreted as correlational; however, it does allow us to determine whether the hypothesised causal pathway is plausible or not. Moreover, path analysis, using longitudinal data, allows us to test causal ordering among sets of variables. We examined the pathways associated with each hypothesised model, which can be summarised as follows:

Model one (M1)—Baseline + controls: childhood SEP + control variables (gender and personality)
Model two (M2)—Psychological model: M1 + self‐efficacy + self‐esteem
Model three (M3)—Exposure model: M1 + parental smoking + household smoking + peer smoking.
Model four (M4)—Social‐resistance model: M1 + experiences of everyday discrimination + levels of opposition to authority.
Model five (M5)—Fully adjusted model.

## RESULTS

**FIGURE 1:**
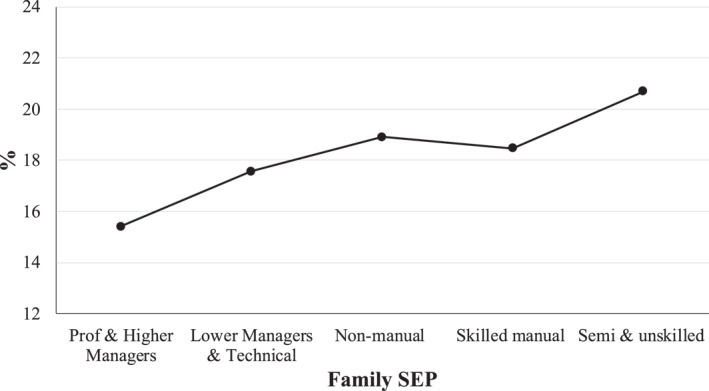
Proportion of smokers by family SEP and gender. SEP, socioeconomic position.

As shown in Figure 1, there is a SEP gradient in adolescent smoking at 17/18, where belonging to a more disadvantaged SEP increases the likelihood of being a smoker. This relationship was only marginally significant (*p* = 0.0587).

**FIGURE 2:**
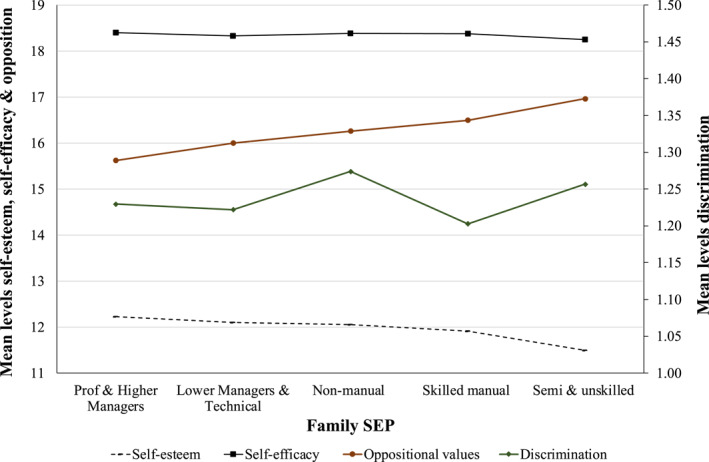
Mean levels of (continuous) mediating variables by family SEP. SEP, socioeconomic position.

**FIGURE 3:**
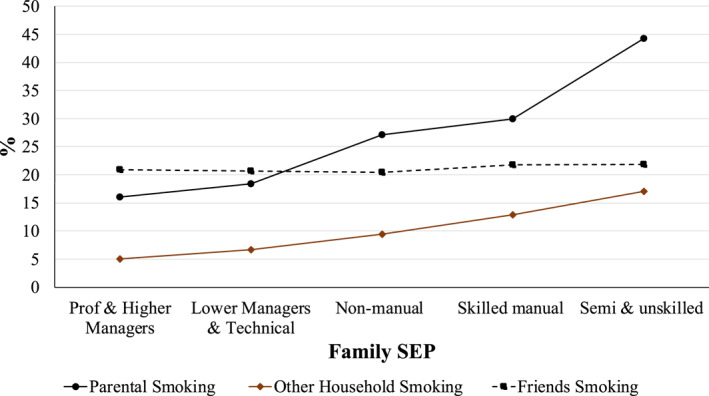
Proportion of (categorical) mediating variables by family SEP. SEP, socioeconomic position.

To descriptively assess whether our mediating processes are adversely distributed by SEP, Figures 2 and 3 provide the means and percentages for each mediating variable by the SEP category. Self‐esteem followed a clear SEP gradient, where belonging to more disadvantaged SEP categories was significantly (*p* < 0.001) associated with decreasing levels of self‐esteem on average. Reported levels of self‐efficacy were not patterned according to SEP, and group differences were not significant. There is a clear SEP gradient in the levels of exposure to smoking from parents (*p* < 0.001) and other household smokers (*p* < 0.001), where young people from more disadvantaged SEP categories were significantly more likely to be exposed to more smoking in the home environment. In terms of exposure to peer smoking, there is no clear SEP gradient, and group differences were not significant. Mean levels of everyday discrimination did not follow any clear SEP patterning, and differences between groups were not significant. However, mean levels of opposition to authority increased, in a stepwise fashion, as SEP decreased, and this relationship was highly significant (*p* < 0.001).

**FIGURE 4:**
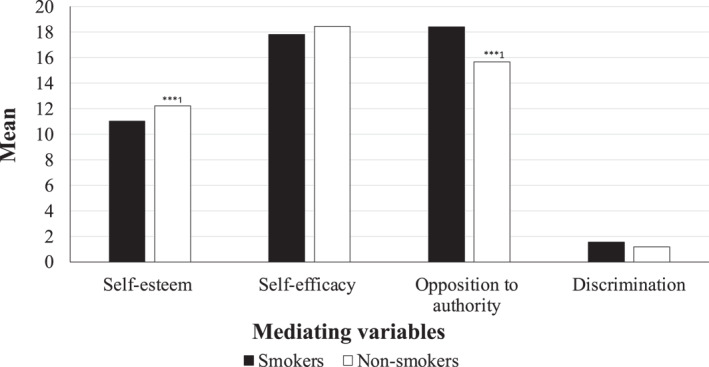
Mean levels of (continuous) mediating variables by smoking status.

**FIGURE 5:**
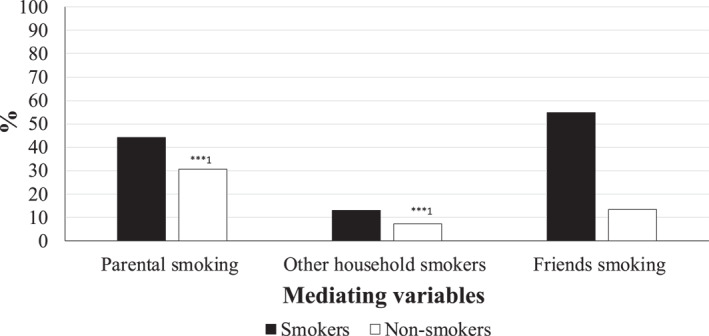
Proportion of (categorical) mediating variables by smoking status.

Do adolescents who smoke have more adverse patterns of our chosen mediating variables compared to those who do not? Figures 4 and 5 provide insight into this by providing means and percentages for the same mediating variables by adolescent smoking status. On average, young people who smoke have significantly higher exposure to smoking from their parents (*p* < 0.001) and other household members (*p* < 0.001), compared to those who do not smoke. Young people who smoke have a significantly higher proportion of smoking friends, compared to those who do not smoke (*p* < 0.001). Young people who smoke, on average, exhibit higher levels of opposition to authority and also tend to have more experiences of everyday discrimination, compared to those who do not smoke (*p* < 0.001). Reported levels of self‐esteem and self‐efficacy were higher amongst for those who do not smoke, compared to those that do smoke (*p* < 0.001).

Do socioeconomically disadvantaged adolescents, with high levels of oppositional values, have more smoking friends? Table 1 confirms that the proportion of friends that smoke increases as levels of oppositional values increase. Of those with high‐levels of oppositional values, young people from the non‐manual households (34.95%) and young people from professionals and higher managerial households (34.26%) report the highest proportion of smoking friends, compared to the other SEP household categories. These results illustrate that the combination of high oppositional values and an advantaged SEP seems to increase the likelihood of having friends who smoke.

**TABLE 1:** Proportion of friends who have smoked by participant oppositional values and SEP.

	Level of oppositional values		
Social class	Low	Medium	High
Prof & higher managers	13.47	19.82	34.26
Lower managers & technical	14.27	20.34	30.66
Non‐manual	11.06	15.71	34.95
Skilled manual	13.80	19.70	31.36
Semi & unskilled	11.81	18.23	31.65

Abbreviation: SEP, socioeconomic position.

**FIGURE 6:**
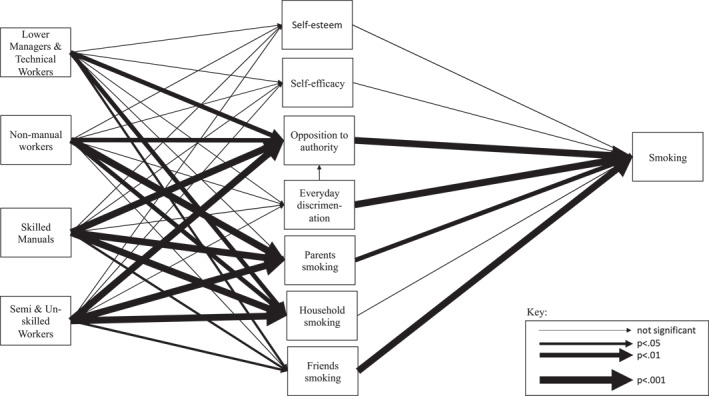
Fully adjusted_2_ GSEM predicting smoking status at age 17/18. GSEM, generalised structural equation modelling.

A total of 6039 subjects were eligible for multivariate analysis. Using GSEM, we tested the ability of our three theoretical models to predict smoking, and the results are displayed graphically in Figure 6 (fully adjusted model) with full tabular results available in the appendix (Table S4). Professionals and higher managerial households are the reference group for family SEP, our independent variable. This category was chosen as it represents the most socioeconomically advantaged families with the lowest proportion of children smoking. Line thickness indicates the level of significance for each of the pathways with thicker lines indicative of more significant relationships, that is, lower *p*‐values. Missing from the figure is the direct pathway from family SEP to smoking, which was removed to simplify the illustration but is included as a pathway in the analyses. Firstly, we tested the variables associated with each model in isolation.

The direct effect of self‐esteem on the probability of smoking was not significant. The direct effect of self‐efficacy on the probability of smoking was also not significant. In short, there was no significant association between the variables that represent the psychological model and family SEP. However, there was a significant (*p* < 0.01) relationship between self‐esteem and smoking status, where low levels of self‐esteem meant the young person was more likely to be a smoker. The combined results demonstrate that there is no significant indirect pathway between SEP and the probability of smoking via our chosen psychological mediating variables, although lower self‐esteem is associated with a higher likelihood of smoking at age 17/18.

Next, we tested the variables associated with the smoking exposure model. The results showed statistically significant direct effects of more disadvantaged SEP on exposure to parental smoking (non‐manual, *p* < 0.001; skilled manual, *p* < 0.001; semi and unskilled, *p* < 0.001) and other household smokers (non‐manual, *p* < 0.01; skilled manual, *p* < 0.001; semi and unskilled, *p* < 0.001). Our results also showed significant direct effects of exposure to parental smoking (*p* < 0.001), and exposure to other household smokers (*p* < 0.01), on the probability of smoking. This acts as evidence for statistically significant mediating pathways between SEP and the probability of smoking via exposure to parental smoking and other household smokers. There was no significant relationship between SEP and being exposed to friends’ smoking. However, the direct effect on exposure to friends smoking and probability of smoking was statistically significant (*p* < 0.001). Taken in unison, this demonstrates that being exposed to friend’s smoking does not mediate the relationship between childhood SEP and smoking status at age 17/18, despite smoking friends’ being strongly associated with a higher likelihood of smoking during this period.

For the social‐resistance model, there were significant direct effects of disadvantaged SEP (lower managers and technical workers, *p* < 0.01; non‐manual, *p* < 0.01; skilled manual, *p* < 0.001; semi and unskilled, *p* < 0.001) on opposition to authority. There were also significant direct effects of opposition to authority (*p* < 0.001), and exposure to everyday discrimination (*p* < 0.001), on the probability of smoking. These results suggest that there are significant mediating pathways between SEP and the probability of smoking via levels of opposition to authority but not via the experiences of discrimination. Furthermore, the direct effect of experiences of discrimination on opposition to authority was also statistically significant (*p* < 0.001). It seems that experiencing discrimination is not a causal pathway through which (1) childhood SEP predicts smoking or (2) childhood SEP results in an increased experience of everyday discrimination which leads to higher levels of opposition to authority, which in turn leads to a higher probability of smoking, as put forward by this paper. However, the experience of discrimination does result in increased oppositional values, on average, as argued by social‐resistance theory.

We used the Akaike information criterion (AIC; Akaike, 1974) and the Bayesian information criterion (BIC; Stone, 1979) to assess which of the three models best fits the observed data. According to these criterion, the smoking exposure model (model 2; AIC = 24,954.26, BIC = 25,279.65) is the superior model in terms of fit, followed by the social‐resistance model (model 3; AIC = 49,805.89, BIC = 50,060.15) with the psychological model (model 1; AIC = 65,535.05, BIC = 65,782.67) explaining the least variation in smoking across our sample.

## DISCUSSION

Although overall prevalence of smoking has declined, social inequalities in smoking continue to increase in adult (Lahelma et al., 2016; Pampel, 2009; WHO, 2022) and youth populations (Rasmussen et al., 2009). This paper confirmed the inverse gradient in smoking, found in previous research, where disadvantaged SEP is associated with a higher likelihood of smoking, in the Irish context, using a nationally representative sample of young people. In the following sections, we have presented the results for our three models, namely: ‘the psychological model,’ ‘the smoking exposure model’ and the ‘social‐resistance model.’ For each model, we theorised how family disadvantage in childhood might lead to smoking in late adolescence, and there is a clear differentiation between individual‐level and structural‐level explanations for inequalities in smoking.

### The psychological model

The psychological model represented an established individual‐level explanation for smoking within the literature. The results of our preliminary bivariate analysis confirmed that self‐esteem and self‐efficacy are important predictors of adolescent smoking, as shown elsewhere (Khosravi et al., 2016; Lawrance and Rubinson, 1986). However, contrary to the underlying argument of the ‘psychological model,’ the results of our bivariate analysis did not show a significant relationship between these psychological traits and family SEP. Furthermore our path analysis demonstrated that neither self‐esteem nor self‐efficacy were mediating pathways between childhood disadvantage and smoking at age 17/18. This suggests that variation in individual psychological traits can aid our understanding of smoking initiation in adolescence, in general, but crucially does not provide insight into how inequalities in smoking emerge during this period.

### The smoking exposure model

Using the same statistical techniques, we found parental smoking to be a statistically significant mediating pathway between childhood disadvantage and smoking at age 17/18, when controlling for all other pathways in the fully adjusted model. Our results revealed that, for young people from disadvantaged families, there was increased exposure to parental smoking, and this exposure increased the likelihood of the young person themselves smoking. Research has previously demonstrated how parental smoking influences the onset of smoking in offspring (Bandura & Walters, 1977; Wellman et al., 2016). We demonstrated how this socialisation process, whereby behaviours are observed and learnt in the home environment, explains differentials in smoking across SEP groups. Alves et al. (2016) also previously found that parental smoking contributes to the transmission of SEP inequalities in adolescent smoking in a sample of students across six European cities.

Being exposed to smoking friends was a strongly significant positive predictor of smoking in adolescence, a relationship that is well‐documented (Simons‐Morton & Farhat, 2010; Wellman et al., 2016). Less is known about whether this relationship differs according to SEP. Exposure to friends’ smoking did not differ significantly by SEP, which suggests that disadvantaged adolescents, although more likely to be smokers themselves, are not more likely to be exposed to more smoking friends than adolescents from more advantaged backgrounds. Harris (2000) argues, in her work, that young people inherit a ‘shared culture’ from their parents that demands the same behaviours, such as smoking. However, our results contradict this idea that young people from disadvantaged backgrounds are more vulnerable to being socialised into smoking via their peer networks. Previous investigation (Moor et al., 2015) on the role of peers in explaining socioeconomic inequalities in adolescent smoking found that ‘peer effects’ are equally important among advantaged and disadvantaged young people and therefore do not contribute to understanding inequalities. It seems that the effects of the peer group and youth culture cut across socioeconomic strata, as theorised by the equalisation hypothesis (West, 1997). In this article, we consider the effect of peer smoking as opposed to quality of peer relationships considered by Moor et al. (2015) for the ‘peer effect’, which may be considered a more direct measure for peer smoking influence. Nonetheless, we find the same results; peer factors did not mediate the relationship between SEP and smoking in adolescence.

### The social resistance model

The results of our path analysis showed that varying levels of oppositional values are a statistically significant mediating pathway between disadvantaged childhood SEP and the probability of smoking at age 17/18, even when controlling for all other pathways in the fully adjusted model. This suggests that the ‘social‐resistance framework’ is a plausible structural explanation for inequalities in smoking in adolescence (Factor et al., 2011). It has been theorised that smoking cessation strategies have an adverse effect in that they strengthen further the social meaning attached to smoking as a physical manifestation of challenging adult authority (Schreuders et al., 2017). Our results seem to support this hypothesis.

In order to contribute to the ongoing debate between the role of ‘peer selection’ and ‘peer socialisation’ processes in the development of substance use in adolescence (Henneberger et al., 2021), we considered the relationship between having smoking friends and oppositional values. Our results showed that young people with high levels of oppositional values had the highest proportion of smoking friends, compared to those that had low or medium levels of oppositional values. Moreover, we found that ‘oppositional’ young people whose parents were ‘non‐manuals’ or ‘professionals’ had the highest proportion of smoking friends. Surprisingly, these young people are some of the least socioeconomically deprived and are also the least likely to be smokers themselves or have smoking parents. We reason that ‘peer selection’ and ‘social‐resistance’ processes work in tandem for these (more advantaged) young people, where they seek out smoking friends as a way to assert an oppositional identity to their non‐smoking parents. In this way, the social resistance process remains the same, however, who they are distinguishing themselves from (Bourdieu, 2018) changes.

Experiencing discrimination was found to be an important predictor of smoking. Moreover, the process, put forward by Factor et al. (2011, 2013) whereby exposure to discrimination prompts ‘resistance,’ was supported here. However, experiences of discrimination were not disproportionately experienced by adolescents from a disadvantaged SEP. Therefore, experiencing discrimination did not prove to mediate the relationship between disadvantaged SEP and smoking initiation. It may be that our measure of discrimination cannot measure the more subtle and nuanced ways in which disadvantaged adolescents experience ‘status anxiety’ (Layte & Whelan, 2014) or feel their own cultural capital is undervalued within society.

### Study limitations

There are limitations to our approach. Our data is concerned with risky (and often illegal) behaviours which may result in potential response bias from young people. We discussed the attempts used to mitigate this risk in the methods section of this paper, which include supplemental self‐complete questionnaires and ensuring the young person of confidentiality.

In terms of measuring the young person’s SEP, more recent literature (Moor et al., 2015) advocates for the use of measures relevant to the age group, for example, young person’s report of their expected educationlevel (Doku et al., 2010; Gagné et al., 2018; LK et al., 2006). For a number of reasons, we did not use this measurement: (1) the advantage of longitudinal panel data meant that we could assess the relationship between childhood SEP and later outcomes. (2) GUI consists of a general measure of expected education‐level, and it has been shown that different dimensions of school‐based status relate to adolescent smoking in opposing directions, meaning that one measure based on several dimensions might show inconsistent relationships (Sweeting & Hunt, 2015). (3) We argue that our measure captures the multidimensionality of SEP, incorporating economic, social, occupational and cultural aspects. Whereas education‐based measures reflect academic attainment and income measures do not incorporate the more nuanced elements of social class (Erikson & Goldthorpe, 1992; Goldthorpe et al., 1987).

Due to the use of smoking as a ‘yes’ or ‘no’ binary variable, it was not possible to carry out path analysis using Structured Equation Modelling (SEM). This study employed GSEM in its place, which has some drawbacks as discussed in the methods section of this paper. Another important limitation related to the use of a simple binary variable for smoking was the lack of specific analysis of ‘occasional smokers,’ which have been shown to display a social gradient (Alves et al., 2023). The proportion of ‘professionals’ that were occasional smokers was less than 5%, that is, too small for further analysis.

We put forward that smoking, as a consumption practice, is an outward material representation of inner feelings of opposition to authority used as a symbolic device to demonstrate distinction. However, we so not wish to imply that this is a deterministic process; a much higher proportion of young people with oppositional values are likely to smoke, compared to young people with low levels of oppositional values, yet, the majority of young people do not smoke at all, which implies that the majority of those with oppositional values still do not smoke.

### Conclusion

This article is an empirical contribution to one of the central problems of sociology: what is the relationship between social structures and individual agency? This article provided empirical evidence that the social context, within which the young person is embedded, has a more important influence on smoking risk than the young person’s individual‐level psychological or personality traits. Individualistic theories, based on human agency, although important for explaining smoking in adolescence, did not explain patterns of smoking behaviour at the aggregate class level. Instead, we found that young people born into disadvantaged households are more likely to have smoking parents, and this exposure makes them more likely to smoke. This would suggest that they are structurally pre‐disposed to smoke. We also found that young people from disadvantaged households have higher levels of oppositional values, compared to their peers from more advantaged households, which is in turn positively associated with smoking initiation. This suggests that smoking risk is determined by one’s positioning in the class structure. However, we argue that individuals possess perceptual boundaries of what is possible and choose symbolic acts of resistance within these boundaries, where outright resistance is not feasible (Scott, 1990). In this way, an economic and social environment produces a socially meaningful behaviour which is inherently concerned with taking back agency.

## AUTHOR CONTRIBUTIONS

**Olivia McEvoy**: Conceptualisation (equal); data curation (lead); formal analysis (lead); investigation (lead); methodology (lead); software (lead); writing—original draft (lead). **Richard Layte**: Conceptualisation (equal); investigation (supporting); methodology (supporting); supervision (lead); writing—review & editing (lead).

## CONFLICT OF INTEREST STATEMENT

The authors declare no conflicts of interest.

## ETHICS STATEMENT

The GUI cohort study, including the materials and procedures adopted at all stages of the study, received ethical approval from an independent Research Ethics Committee set up by the Department of Children, Equality, Disability, Integration and Youth.

## Supporting information

Supporting Information S1

## Data Availability

The data that supports the findings of this paper is from the Restricted Microdata Files (RMF) of Growing Up in Ireland (GUI) cohort study, available from the Central Statistics Office (CSO). All requests for access to RMFs, from the CSO, are made through the Researcher Online System for Applications (ROSA). Only researchers who are employed by, or formally related to, a registered research organisation will be eligible to apply for access to RMFs. Researchers wishing to use the RMFs need to complete training, on ROSA, to become an Officer of Statistics and make an official application for access, detailing the aims and objectives of the research paper. In order for RMF access to be granted, approval must be granted by a Statistician, a Senior Statistician, an Assistant Director General and by the Director General of the CSO. Researchers seeking to use GUI anonymised data must agree to these conditions and abide by any other conditions, such as relating to data security, as set out by the Central Statistics Office, DCEDIY, ISSDA, the GUI Study Team or related bodies.
