# Supplementary material for: Bringing the group back in: Social class and resistance in adolescent smoking
Source: Sociol Health Illn. 2024 Oct 22;47(2):e13858. doi: 10.1111/1467-9566.13858 (PMC11849772; doi:10.1111/1467-9566.13858)
Supplement: Supplementary file 1 — Supporting Information S1 [file SHIL-47-0-s001.docx]

# Appendix

# List of abbreviations

SEP Socio Economic Position

NCD Non-Communicable Diseases

WHO World health Organisation

HSE Health Service Executive

OECD Organisation for Economic Co-operation and Development

GUI Growing Up in Ireland

MICE Multiple Imputation by Chained Equations

CSO Central Statistics Office

PCG Primary Caregiver

SCG Secondary Caregiver

EDS Everyday Discrimination Scale

ESRC Economic and Social Research Council

TIPI Ten-Item Personality Inventory

CI Confidence Interval

GSEM Generalised Structural Equation Modelling

SEM Structural Equation Modelling

AIC Akaike Information Criterion

BIC Bayesian Information Criterion

# Supplementary Tables

Table 1: Proportion of missing values.

| Dependent variable | % missing |
| --- | --- |
| Smoking status at 17 | 1.09% |
| Treatment variable |  |
| Family SEP | 0.07% |
| Control variables |  |
| Gender | 0.00% |
| Extraversion | 0.13% |
| Agreeableness | 0.07% |
| Conscientiousness | 0.09% |
| Emotional stability | 0.09% |
| Openness to experience | 0.19% |
| Mediating variables |  |
| *Psychological model* |  |
| Self-esteem | 1.43% |
| Self-efficacy | 1.35% |
| *Exposure model* |  |
| Parental smoking | 2.43% |
| Other household members smoking | 2.43% |
| Friends smoking | 0.00% |
| *Social resistance model* |  |
| Everyday discrimination | 1.24% |
| Opposition to authority | 3.68% |

Table 2. Descriptive statistics for mediating variables by family social class.

|  |  | Five Household Social Class Categories | | | | | | | | | | | |
| --- | --- | --- | --- | --- | --- | --- | --- | --- | --- | --- | --- | --- | --- |
|  |  | Professionals & Higher  Managers | | Lower Manager &  Technical | | Non-manual | | Skilled  Manual | | Semi &  Unskilled | |  | |
|  |  | (N=1172) | | (N=3317) | | (N=1686) | | (N=1196) | | (N=1191) | |  | |
|  |  | Mean | (SD) | Mean | (SD) | Mean | (SD) | Mean | (SD) | Mean | (SD) | *p value* |  |
|  | *Exogenous Variables* |  |  |  |  |  |  |  |  |  |  |  |  |
| **Psychological Model** | Self-esteem | 12.22 | 3.58 | 12.10 | 3.48 | 12.05 | 3.48 | 11.91 | 3.54 | 11.49 | 3.69 | <.001 |  |
|  | Self-efficacy | 18.40 | 2.74 | 18.33 | 2.75 | 18.38 | 2.88 | 18.38 | 2.81 | 18.25 | 2.81 | 0.80 |  |
| **Social Resistance Model** | Opposition to authority | 15.62 | 3.41 | 16.00 | 3.56 | 16.26 | 3.60 | 16.49 | 3.59 | 16.97 | 3.74 | <.001 |  |
|  | Everyday discrimination | 1.23 | 0.78 | 1.22 | 0.83 | 1.27 | 0.89 | 1.20 | 0.89 | 1.26 | 0.93 | 0.33 |  |
|  |  | N | % | N | % | N | % | N | % | N | % |  |  |
| **Smoking Exposure Model** | Parent's smoking | 172 | 16.06 | 549 | 18.42 | 386 | 27.09 | 300 | 29.94 | 429 | 44.27 | <.001 |  |
|  | Other household smokers | 54 | 5.05 | 200 | 6.72 | 134 | 9.42 | 129 | 12.91 | 165 | 17.1 | <.001 |  |
|  | Friends smoking | 195 | 20.9 | 516 | 20.66 | 236 | 20.43 | 176 | 21.81 | 162 | 21.83 | 0.91 |  |

Table 3. Descriptive statistics for mediating variables for smokers and non-smokers.

|  |  | Smoking status at 17 | | | | |
| --- | --- | --- | --- | --- | --- | --- |
|  |  | Smokers | | Non-smokers | |  |
|  |  | (N=1118) | | (N=5098) | |  |
|  |  | Mean | (SD) | Mean | (SD) | *p value* |
|  | *Exogenous Variables* |  |  |  |  |  |
| **Psychological Model** | Self-esteem | 11.04 | 0.11 | 12.23 | 0.05 | <.001 |
|  | Self-efficacy | 17.81 | 0.08 | 18.46 | 0.04 | <.001 |
| **Social Resistance Model** | Opposition to authority | 18.42 | 0.11 | 15.67 | 0.05 | <.001 |
|  | Everyday discrimination | 1.55 | 0.03 | 1.16 | 0.01 | <.001 |
|  |  | N | % | N | % | *p value* |
| **Smoking Exposure Model** | Parent's smoking | 352 | 32.77 | 1067 | 21.7 | <.001 |
|  | Other household smokers | 140 | 13.06 | 358 | 7.29 | <.001 |
|  | Friends smoking | 1091 | 13.46 | 3825 | 54.72 | <.0.01 |

Table 4. Path coefficients of exogenous and endogenous variables estimated using logistic regression as part of generalised structural equation modelling.

|  |  | Controls Model (N=6039) | | Psychological Model (N=6039) | | Exposure model (N=6039) | | Resistance Model (N=6039) | | Fully Adjusted Model (N=6039) | |
| --- | --- | --- | --- | --- | --- | --- | --- | --- | --- | --- | --- |
|  |  | coef . | S.E | coef . | S.E | coef . | S.E | coef . | S.E | coef . | S.E |
| Predicting smoking  status | female | -0.13 | 0.09 | 0.02 | 0.10 | -0.09 | 0.10 | -0.34** | 0.10 | -0.25* | 0.11 |
|  | Lower Manager & Technical  (vs. professional) | 0.09 | 0.16 | 0.09 | 0.16 | 0.06 | 0.16 | 0.00 | 0.16 | -0.01 | 0.17 |
|  | Non-manuals  (vs. professionals) | 0.23 | 0.17 | 0.21 | 0.16 | 0.17 | 0.19 | 0.05 | 0.18 | 0.04 | 0.19 |
|  | Skilled manuals  (vs. professionals) | 0.17 | 0.18 | 0.18 | 0.18 | 0.06 | 0.19 | -0.00 | 0.19 | -0.05 | 0.20 |
|  | Semi & unskilled  (vs. professionals) | 0.23 | 0.18 | 0.20 | 0.18 | 0.10 | 0.20 | -0.07 | 0.19 | -0.11 | 0.21 |
|  | Extraversion | 0.075699** | 0.02 | 0.09** | 0.03 | 0.05 | 0.03 | 0.07* | 0.03 | 0.05 | 0.03 |
|  | Agreeableness | -0.11*** | 0.02 | -0.11*** | 0.03 | -0.09** | 0.03 | -0.07* | 0.03 | -0.07* | 0.03 |
|  | Conscientiousness | -0.13*** | 0.02 | -0.12*** | 0.24 | -0.11*** | 0.03 | -0.10*** | 0.02 | -0.09** | 0.03 |
|  | Emotional stability | 0.01 | 0.02 | 0.02 | 0.03 | 0.01 | 0.03 | 0.01 | 0.03 | 0.02 | 0.03 |
|  | Openness to experience | 0.03 | 0.03 | 0.03 | 0.03 | 0.02 | 0.03 | 0.03 | 0.03 | 0.02 | 0.03 |
|  | Self-esteem |  |  | -0.07*** | 0.02 |  |  |  |  | -0.02 | 0.02 |
|  | Self-efficacy |  |  | -0.02 | 0.02 |  |  |  |  | 0.01 | 0.02 |
|  | Parental smokers |  |  |  |  | 0.45*** | 0.12 |  |  | 0.39** | 0.12 |
|  | Household smokers |  |  |  |  | 0.14* | 0.07 |  |  | 0.12 | 0.07 |
|  | Friend smokers |  |  |  |  | 1.96*** | 0.10 |  |  | 1.75*** | 0.11 |
|  | Opposition to authority |  |  |  |  |  |  | 0.18*** | 0.02 | 0.14*** | 0.02 |
|  | Experience everyday discrimination |  |  |  |  |  |  | 0.23*** | 0.06 | 0.18* | 0.07 |
|  | Constant | -0.93 | 0.24 | 0.10 | 0.36 | -1.74 | 0.27 | -4.28 | 0.36 | -4.40 | 0.56 |
| Predicting levels of self-esteem | female |  |  | 1.95*** | 0.12 |  |  |  |  | 1.96*** | 0.12 |
|  | Lower Manager & Technical  (vs. professional) |  |  | 0.00 | 0.17 |  |  |  |  | 0.01 | 0.17 |
|  | Non-manuals  (vs. professionals) |  |  | -0.17 | 0.20 |  |  |  |  | -0.18 | 0.20 |
|  | Skilled manuals  (vs. professionals) |  |  | -0.05 | 0.21 |  |  |  |  | -0.04 | 0.21 |
|  | Semi & unskilled  (vs. professionals) |  |  | -0.41 | 0.22 |  |  |  |  | -0.37 | 0.22 |
|  | Extraversion |  |  | 0.07* | 0.03 |  |  |  |  | 0.07 | 0.03 |
|  | Agreeableness |  |  | 0.04 | 0.03 |  |  |  |  | 0.04* | 0.03 |
|  | Conscientiousness |  |  | 0.10** | 0.03 |  |  |  |  | 0.10** | 0.03 |
|  | Emotional stability |  |  | 0.15*** | 0.03 |  |  |  |  | 0.15*** | 0.03 |
|  | Openness to experience |  |  | -0.04 | 0.03 |  |  |  |  | -0.04 | 0.04 |
|  | Constant |  |  | 9.76 | 0.30 |  |  |  |  | 9.74 | 0.29 |
| Predicting levels of self-efficacy | female |  |  | 0.58*** | 0.10 |  |  |  |  | 0.58*** | 0.10 |
|  | Lower Manager & Technical  (vs. professional) |  |  | 0.14 | 0.15 |  |  |  |  | 0.14 | 0.15 |
|  | Non-manuals  (vs. professionals) |  |  | 0.04 | 0.17 |  |  |  |  | 0.04 | 0.17 |
|  | Skilled manuals  (vs. professionals) |  |  | 0.28 | 0.18 |  |  |  |  | 0.28 | 0.18 |
|  | Semi & unskilled  (vs. professionals) |  |  | 0.11 | 0.18 |  |  |  |  | 0.11 | 0.18 |
|  | Extraversion |  |  | 0.14* | 0.03 |  |  |  |  | 0.14*** | 0.03 |
|  | Agreeableness |  |  | 0.02 | 0.03 |  |  |  |  | 0.02 | 0.03 |
|  | Conscientiousness |  |  | 0.06** | 0.03 |  |  |  |  | 0.06* | 0.03 |
|  | Emotional stability |  |  | 0.09*** | 0.03 |  |  |  |  | 0.09*** | 0.03 |
|  | Openness to experience |  |  | 0.02 | 0.03 |  |  |  |  | 0.02 | 0.03 |
|  | Constant |  |  | 16.57 | 0.24 |  |  |  |  | 16.57 | 0.24 |
| Predicting exposure to  parents smoking | female |  |  |  |  | -0.12 | 0.08 |  |  | -0.12 | 0.08 |
|  | Lower Manager & Technical  (vs. professional) |  |  |  |  | 0.16 | 0.13 |  |  | 0.16 | 0.13 |
|  | Non-manuals  (vs. professionals) |  |  |  |  | 0.73*** | 0.14 |  |  | 0.73*** | 0.14 |
|  | Skilled manuals  (vs. professionals) |  |  |  |  | 0.93*** | 0.14 |  |  | 0.93*** | 0.14 |
|  | Semi & unskilled  (vs. professionals) |  |  |  |  | 1.24*** | 0.15 |  |  | 1.24*** | 0.15 |
|  | Extraversion |  |  |  |  | 0.03 | 0.02 |  |  | 0.03 | 0.02 |
|  | Agreeableness |  |  |  |  | -0.03 | 0.02 |  |  | -0.03 | 0.02 |
|  | Conscientiousness |  |  |  |  | -0.06** | 0.02 |  |  | -0.06** | 0.02 |
|  | Emotional stability |  |  |  |  | -0.02 | 0.02 |  |  | -0.02 | 0.02 |
|  | Openness to experience |  |  |  |  | -0.02 | 0.04 |  |  | 0.02 | 0.02 |
|  | Constant |  |  |  |  | -0.79 | 0.19 |  |  | -0.79 | 0.19 |
| Predicting exposure to  smokers in the household (excluding parents) | female |  |  |  |  | -0.13 | 0.12 |  |  | -0.13 | 0.12 |
|  | Lower Manager & Technical  (vs. professional) |  |  |  |  | 0.21 | 0.25 |  |  | 0.21** | 0.25 |
|  | Non-manuals  (vs. professionals) |  |  |  |  | 0.71** | 0.27 |  |  | 0.71** | 0.27 |
|  | Skilled manuals  (vs. professionals) |  |  |  |  | 0.97*** | 0.26 |  |  | 0.97*** | 0.26 |
|  | Semi & unskilled  (vs. professionals) |  |  |  |  | 1.27*** | 0.26 |  |  | 1.27*** | 0.26 |
|  | Extraversion |  |  |  |  | 0.09* | 0.03 |  |  | 0.09 | 0.03 |
|  | Agreeableness |  |  |  |  | -0.04 | 0.03 |  |  | -0.04*** | 0.03 |
|  | Conscientiousness |  |  |  |  | -0.06* | 0.03 |  |  | -0.06*** | 0.03 |
|  | Emotional stability |  |  |  |  | -0.00 | 0.04 |  |  | 0.00 | 0.03 |
|  | Openness to experience |  |  |  |  | -0.02 | 0.04 |  |  | -0.02 | 0.04 |
|  | Constant |  |  |  |  | -2.45 | 0.30 |  |  | -2.45 | 0.30 |
| Predicting exposure to smoking  friends | female |  |  |  |  | -0.02 | 0.09 |  |  | -0.02 | 0.09 |
|  | Lower Manager & Technical  (vs. professional) |  |  |  |  | -0.01 | 0.14 |  |  | -0.01 | 0.14 |
|  | Non-manuals  (vs. professionals) |  |  |  |  | -0.29 | 0.15 |  |  | -0.03 | 0.15 |
|  | Skilled manuals  (vs. professionals) |  |  |  |  | -0.06 | 0.16 |  |  | 0.06 | 0.16 |
|  | Semi & unskilled  (vs. professionals) |  |  |  |  | -0.03 | 0.16 |  |  | 0.09 | 0.17 |
|  | Extraversion |  |  |  |  | 0.09*** | 0.02 |  |  | 0.09*** | 0.02 |
|  | Agreeableness |  |  |  |  | -0.06* | 0.02 |  |  | -0.06** | 0.02 |
|  | Conscientiousness |  |  |  |  | -0.06 | 0.02 |  |  | -0.06** | 0.02 |
|  | Emotional stability |  |  |  |  | -0.02 | 0.02 |  |  | -0.02 | 0.02 |
|  | Openness to experience |  |  |  |  | 0.02 | 0.03 |  |  | 0.03 | 0.03 |
|  | Constant |  |  |  |  | -1.01 | 0.21 |  |  | -1.01 | 0.21 |
| Predicting levels of  opposition to authority | female |  |  |  |  |  |  | 0.67*** | 0.08 | 0.66*** | 0.13 |
|  | Lower Manager & Technical  (vs. professional) |  |  |  |  |  |  | 0.57** | 0.19 | 0.53** | 0.18 |
|  | Non-manuals  (vs. professionals) |  |  |  |  |  |  | 0.75** | 0.22 | 0.72** | 0.22 |
|  | Skilled manuals  (vs. professionals) |  |  |  |  |  |  | 1.07*** | 0.23 | 1.02*** | 0.23 |
|  | Semi & unskilled  (vs. professionals) |  |  |  |  |  |  | 1.58*** | 0.24 | 1.55*** | 0.23 |
|  | Extraversion |  |  |  |  |  |  | -0.00 | 0.03 | 0.00 | 0.03 |
|  | Agreeableness |  |  |  |  |  |  | -0.14*** | 0.04 | -0.14*** | 0.03 |
|  | Conscientiousness |  |  |  |  |  |  | -0.14*** | 0.03 | -0.14*** | 0.03 |
|  | Emotional stability |  |  |  |  |  |  | 0.01 | 0.03 | 0.00 | 0.03 |
|  | Openness to experience |  |  |  |  |  |  | 0.02 | 0.04 | 0.01 | 0.04 |
|  | Everyday discrimination |  |  |  |  |  |  | 1.44*** | 0.08 | 1.43*** | 0.08 |
|  | Constant |  |  |  |  |  |  | 14.66 | 0.35 | 14.72 | 0.34 |
| Predicting experiencing everyday acts of discrimination | female |  |  |  |  |  |  | 0.12*** | 0.03 | 0.12*** | 0.03 |
|  | Lower Manager & Technical  (vs. professional) |  |  |  |  |  |  | -0.05 | 0.05 | -0.05 | 0.05 |
|  | Non-manuals  (vs. professionals) |  |  |  |  |  |  | 0.04 | 0.06 | 0.04 | 0.06 |
|  | Skilled manuals  (vs. professionals) |  |  |  |  |  |  | -0.07 | 0.06 | -0.06 | 0.06 |
|  | Semi & unskilled  (vs. professionals) |  |  |  |  |  |  | -0.00 | 0.06 | -0.00 | 0.06 |
|  | Extraversion |  |  |  |  |  |  | 0.03* | 0.01 | 0.27** | 0.01 |
|  | Agreeableness |  |  |  |  |  |  | -0.04*** | 0.01 | -0.04*** | 0.01 |
|  | Conscientiousness |  |  |  |  |  |  | -0.03*** | 0.01 | -0.03*** | 0.01 |
|  | Emotional stability |  |  |  |  |  |  | -0.02 | 0.01 | -0.02 | 0.01 |
|  | Openness to experience |  |  |  |  |  |  | 0.01 | 0.01 | 0.01 | 0.01 |
|  | Constant |  |  |  |  |  |  | 1.46 | 0.07 | 1.46 | 0.07 |

***p<.05, ***p<.01, ***p<.001**
